# Effects of prior anterior cruciate ligament reconstruction on clinical outcomes associated with total knee arthroplasty: A protocol of retrospective analysis

**DOI:** 10.1097/MD.0000000000020767

**Published:** 2020-06-19

**Authors:** Li-Zuo Wang, Ai-Hua Liu, De-Jian Wen, Yan-Tao Zhou

**Affiliations:** a Department of Orthopedics and Traumatology; b Department of Orthopedics, the Central Hospital of Enshi Tujia and Miao Autonomous Prefecture; c Department of Medicine, Hubei Minzu University, Hubei, China.

**Keywords:** anterior cruciate ligament reconstruction, complication, study protocol, total knee arthroplasty

## Abstract

**Background:**

The argument on the clinical effects of previous anterior cruciate ligament (ACL) reconstruction on total knee arthroplasty (TKA) remains to be resolved. The aim of the current study was to compare operative and postoperative outcomes of patients undergoing TKA after ACL reconstruction with a matched cohort of control subjects having primary osteoarthritis and no history of ligament reconstruction.

**Methods:**

This study was performed and reported in accordance with the Strengthening the Reporting of Observational studies in Epidemiology checklist. The institutional review board approval of our hospital was obtained for the study. The ACL and control groups were matched 1:1 using a caliper width of 0.1 for the propensity score through nearest neighbor matching. Written informed consent was obtained from all subjects participating in the trial. The primary outcome measure was postoperative complications. Secondary outcome measures included operative time, tourniquet time, intraoperative complications, Oxford Knee Score, range of motion, and Western Ontario and McMaster Universities index.

**Results:**

This study had limited inclusion and exclusion criteria and a well-controlled intervention. We hypothesized that prior ACL reconstruction had a negative impact on the operative and postoperative outcomes of TKA.

Trial registration: This study protocol was registered in Research Registry (researchregistry5598).

## 1 Introduction

The rupture of anterior cruciate ligament (ACL) is one of the most common knee injuries among the young, active population, with more than 200,000 cases estimated to occur in the United States every year.^[1]^ If left untreated, the injury of ACL often results in chronic knee instability, which may contribute to secondary injuries such as knee osteoarthritis and meniscal tears.^[2–4]^ Although nonoperative treatment has been proven successful in short term after ACL rupture,^[5,6]^ ACL reconstruction is usually regarded as the gold standard in treatment of ACL rupture, with approximately 175,000 reconstructions performed annually.^[7]^ The aim of the reconstruction is to restore knee laxity to normal levels and thus restore its stability and improve function. Nevertheless, a subsequent decrease in the rate of osteoarthritis development following ACL rupture has not been demonstrated, irrespective of whether the patients were treated operatively or nonoperatively.^[8]^

Total knee arthroplasty (TKA) is considered to be an effective treatment for patients with knee osteoarthritis. Brophy et al, in a study with 1372 patients reviewed, demonstrated that patients with a history of ligament reconstruction underwent TKA at a significantly younger age than patients with or without a history of other knee surgery.^[7]^ With dramatically increasing number of ACL reconstructions and TKA performed in United States yearly, surgeons can expect to see increasing numbers of patients with a history of ACL reconstruction. Therefore, the question of whether the previous ACL reconstruction will adversely affect subsequent TKA is very meaningful.

To our knowledge, the argument on the clinical effects of previous ACL reconstruction on TKA remains to be resolved. Additionally, several recent retrospective studies have reported it with conflicting results. Limited by sample size, these studies failed to show a clear conclusion.^[9,10]^^11–13^ Therefore, the aim of the current study was to compare operative and postoperative outcomes of patients undergoing TKA after ACL reconstruction with a matched cohort of control subjects having primary osteoarthritis and no history of ligament reconstruction. We hypothesized that prior ACL reconstruction had a negative impact on the operative and postoperative outcomes of TKA.

## 2 Materials and methods

### 2.1 Study design

We retrospectively identified 2310 patients who underwent primary TKA at our academic institution between 2010 and 2014 from our registry database. The Institutional Review Board of the Central Hospital of Enshi approved the study protocol (IRB: ES370042). Written informed consent was obtained from all subjects participating in the trial. This study was also registered in the research registry (researchregistry5598). This study was performed and reported in accordance with the Strengthening the Reporting of Observational studies in Epidemiology checklist.

### 2.2 Inclusion and exclusion criteria

The inclusion criterion were set as follows:

(1) osteoarthritis of the knee requiring primary TKA during hospitalization;
(2) patients that were over 18 years old and could cooperate with us for treatment and postoperative observation;
(3) full demographic and follow-up data. Subjects with a history of inflammatory arthropathy, traumatic surgery, previous fracture, and history of surgery that might increase osteoarthritis risk, such as lower extremity osteotomy and fracture fixation about the knee, were excluded from the study.

### 2.3 Propensity score matching

We used the propensity score-matching method to adjust the baseline differences between the ACL and control groups in an effort to derive more accurate conclusions. Multivariate logistic regression was used to determine propensity scores for each patient based on age, sex, body mass index, and the American Society of Anesthesiologists score, which were demographics before propensity score-matching between the 2 groups. ACL and control were matched 1:1 using a caliper width of 0.1 for the propensity score through nearest neighbor matching.

### 2.4 Surgical techniques

All operations were performed under general anesthesia in an operating room with laminar flow. The surgical technique and instrumentation were similar in all knees. All operations performed during the study period in both the knee ligament reconstruction and control groups were performed by the same arthroplasty surgeons. A tourniquet was used in all cases, and the operative knee was prepared and draped in a conventional sterile fashion. The implants utilized were beaded periapatite-coated femoral, tibial, and patellar components (Triathlon Total Knee System; Stryker Orthopaedics, Mahwah, NJ).

### 2.5 Outcome measures

The primary outcome measure was postoperative complications such as infection, aseptic loosening, wound complication, manipulation under anesthesia, and contracture. Secondary outcome measures included operative time (time from incision to completion of skin closure), tourniquet time, intraoperative complications, Oxford Knee Score (OKS), range of motion, and Western Ontario and McMaster Universities index (WOMAC).

Complications, operative time, and tourniquet time were documented during routine collection of follow-up data. All data were independently verified by a detailed review of hospital operative reports, anesthesia records, and clinical records. The OKS, range of motion, and WOMAC were obtained both before and after surgery at a minimum of 5 years postoperatively. Subjects were asked to complete OKS and WOMAC questionnaire and rate their satisfaction with their TKA. The range of motion were measured by the research assistant for both knees with a standard universal goniometer, which has been shown to have good intraobserver and interobserver reliability in the knee joint.

### 2.6 Statistical analysis

Statistical analyses were conducted using SPSS v22.0 software (IBM, Chicago, IL). Descriptive statistics were utilized to compare the demographic characteristics of both groups, as well as clinical hip scores and follow-up duration. Statistical analysis included the Student *t* test for continuous variables and the Fisher exact test for categorical variables. All probability values were 2-tailed, with *P* < .05 regarded as statistically significant.

## 3 Results

The results will be shown in Tables 1–3.

**Table 1:**
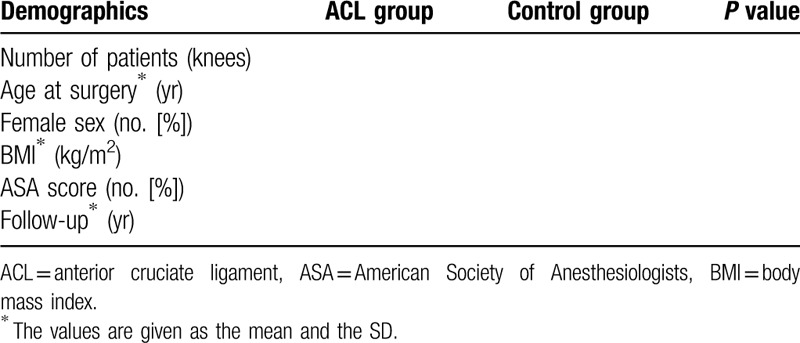
Patient baseline demographics.

**Table 2:**
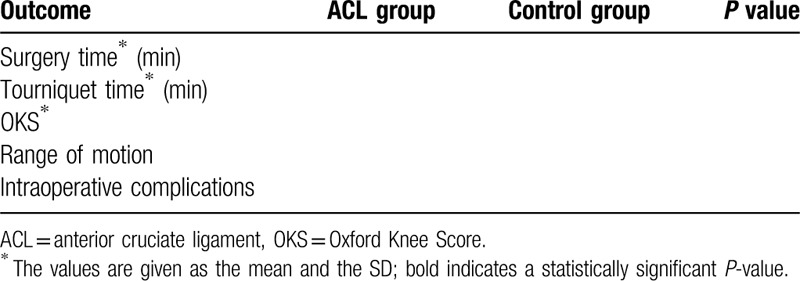
Intraoperative and functional outcomes.

**Table 3:**
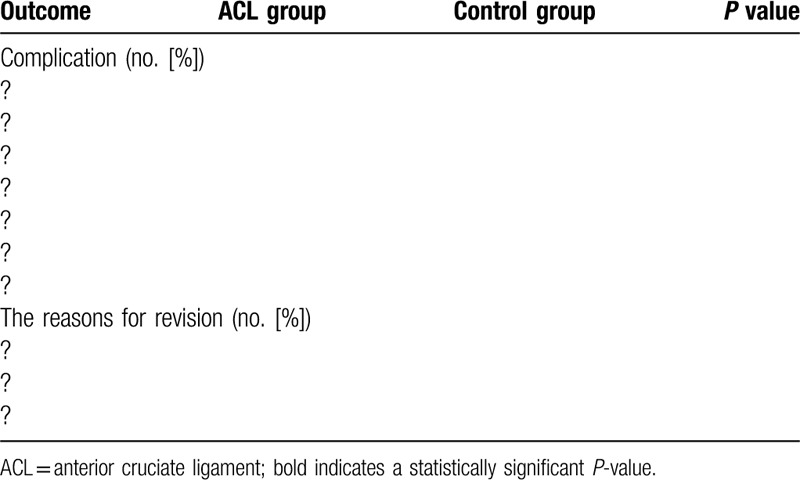
The complications and revision in the 2 groups.

## 4 Discussion

It is estimated that more than 175,000 ACL reconstructions are performed annually in the United States,^[7]^ with a recent epidemiology study suggesting an early 50% increase in the incidence of ACL reconstruction over a 12-year period from 1994 to 2006.^[9]^ Consequently, patients with a history of a knee surgical procedure, especially men and those with a history of ligament reconstruction, have been shown to undergo TKA at a younger age than patients without a history of a knee surgical procedure.^[10]^ As the number of patients requiring TKA in the United States continues to dramatically increase, orthopedic surgeons can expect to see increasing numbers of patients with a history of ACL reconstruction. Furthermore, few studies in literature has studied the outcomes of TKA after ACL reconstruction. Therefore, the purpose of this study was to compare operative characteristics and early outcomes of a large series of patients undergoing TKA after ACL reconstruction with a matched cohort of control subjects having primary osteoarthritis and no history of ligament reconstruction.

The limitations of our study included those inherent in any retrospective cohort study, including the possibility of selection or observational bias. This study also did not address long-term follow-up (15 years) as our study relied on electronic medical records kept since 2010. The authors recognize that longer term follow-up is critical in determining the influence of prior ACL reconstruction on TKA specifically on infection, implant loosening, revision, and long-term function outcomes. Additionally, although we performed a matched study based on age, gender, American Society of Anesthesiologists score, and body mass index, it is likely that there were other pre-operative features that we could have controlled for that may have led to alternative results.

## Author contributions

**Conceptualization:** Li-zuo Wang, Ai-hua Liu.

**Data curation:** Li-zuo Wang, Ai-hua Liu.

**Formal analysis:** Li-zuo Wang, Ai-hua Liu.

**Funding acquisition:** Yan-tao Zhou.

**Investigation:** Li-zuo Wang, Ai-hua Liu.

**Methodology:** De-jian Wen.

**Resources:** Yan-tao Zhou.

**Software:** De-jian Wen.

**Supervision:** Yan-tao Zhou.

**Validation:** De-jian Wen.

**Visualization:** De-jian Wen.

**Writing – original draft:** Li-zuo Wang, Ai-hua Liu

**Writing – review & editing:** Yan-tao Zhou.
